# The Antifungal Activity of Loquat (*Eriobotrya japonica* Lindl.) Leaves Extract Against *Penicillium digitatum*

**DOI:** 10.3389/fnut.2021.663584

**Published:** 2021-08-20

**Authors:** Yuting Shen, Chuying Chen, Nan Cai, Ruopeng Yang, Jinyin Chen, İbrahim Kahramanoǧlu, Volkan Okatan, Kannan R. R. Rengasamy, Chunpeng Wan

**Affiliations:** ^1^Jiangxi Key Laboratory for Postharvest Technology and Nondestructive Testing of Fruits, Vegetables/Collaborative Innovation Center of Postharvest Key Technology and Quality Safety of Fruits and Vegetables in Jiangxi Province, College of Agronomy, Jiangxi Agricultural University, Nanchang, China; ^2^College of Materials and Chemical Engineering, Pingxiang University, Pingxiang, China; ^3^Faculty of Agricultural Sciences and Technologies, European University of Lefke, Gemikonagi, Turkey; ^4^Department of Horticulture, Faculty of Agriculture, Eskişehir Osmangazi University, Eskişehir, Turkey; ^5^Green Biotechnologies Research Centre of Excellence, University of Limpopo, Mankweng, South Africa

**Keywords:** loquat leaves, *Penicillium digitatum*, membrane permeability, energy metabolism, antifungal activity

## Abstract

This study was performed to determine the antifungal activity of loquat (*Eriobotrya japonica* Lindl) leaf extract (LLE) against the citrus postharvest pathogen *Penicillium digitatum* (*P. digitatum*). The LLE exhibited an antifungal activity against *P. digitatum*, with a minimum inhibitory concentration (MIC) of 0.625 mg/ml and a minimum fungicidal concentration (MFC) of 1.25 mg/ml. Significant inhibitory effects of LLE on mycelial growth and spore germination of *P. digitatum* were seen in a dose-dependent manner. Simultaneously, to investigate possible antifungal mechanisms by LLE, we analyzed their influence on morphological changes, cell membrane permeability, cell wall and cell membrane integrity, and adenosine phosphates (ATP, ADP, and AMP) levels. Alterations, such as sunken surface and malformation, occurred in the LLE-treated *P. digitatum* spores. Furthermore, intracellular inclusion content decreased after LLE treatment, indicating an increase in cell membrane permeability. Besides, the LLE treatment induced a significant decline in the level of adenosine monophosphate (AMP), adenosine diphosphate (ADP), and adenosine triphosphate (ATP) with a noticeable addition of extracellular ATP, ADP, and AMP during the entire treatment period. Overall, the results manifested that the antifungal activity of LLE against *P. digitatum* can be attributed to the derangement of cell membrane permeability and disordered energy metabolism. This is the first report on the mechanism of antifungal activity of LLE and could be useful in the development of targeted fungicides from natural origin.

## Introduction

Massive postharvest losses in citrus fruits during storage, transportation, and selling are mainly caused by green (*Penicillium digitatum*) and blue (*Penicillium italicum*) mold fungus. Moreover, the sour rot and stem-end rot diseases caused by *Geotrichum citri-aurantii* and *Diaporthe citri*, respectively, may also contribute to postharvest losses in citrus fruits (1). *P. digitatum* is the most common pathogen reported to have caused about 90% of total postharvest loss in citrus fruits (2). Currently, postharvest fungal diseases are controlled and prevented using chemical fungicides, such as imazalil, prochloraz, thiabendazole, and many others (3–5). However, excessive use of chemical fungicides causes environmental problems and potential health issues in humans and animals. This also leads to the development of resistant fungal strains, which leads to devastating results. Therefore, the current demand is to explore and develop natural and effective antifungal agents as alternatives to chemical fungicides.

Plant-derived extracts, such as essential oils, are generally recognized as safe (GRAS) components (6). The application of several plant extracts (e.g., pomegranate peel, pompia leave, *Ficus hirta* Vahl. Fruit, and *Sapindus saponaria* L. fruit) has been reported to reduce postharvest fungal diseases in citrus fruits and other horticultural commodities (7–10). For instance, the essential oil extracted from pompia leaves effectively controlled the growth of *P. digitatum* (8). Moreover, the results showed that the activity of the ethanol extract of *Sapindus saponaria* L. fruit against *Colletotrichum Musae* was similar to that of thiabendazole at a 500-μg/ml concentration (10). Pomegranate peel extract was also very effective against plant diseases by inducing and enriching the fruit critical defense pathways and antibiotic biosynthesis (7). Also, postharvest fruit loss due to fungal pathogen during storage was effectively controlled by the *F. hirta* Vahl. fruit extract (9).

Loquat (*Eriobotrya japonica* Lindl) is a subtropical perennial fruit tree widely distributed in southeastern China and highly consumed because of its soft and juicy pulp, delicious taste, and health-related properties (11). Moreover, the leaves of loquat, commonly known by the name of “Pí Pá Yè” in Chinese pharmacopeia, possess enormous biological activities and are extensively used to treat cough ailments and pulmonary diseases, chronic bronchitis, inflammation, and diabetes (11, 12). The leaves of loquat are particularly rich in phenolics and have a potent antioxidant activity (12, 13). To date, no information is available on the antifungal effect of loquat leaf extract on postharvest citrus fruits against fungal pathogens. Therefore, this work aimed to investigate the *in vitro* antifungal activity of loquat leaf extract (LLE) on postharvest citrus fruit pathogens, namely, *P. digitatum, P. italicum, D. citri*, and *G. citri-auranti*. Moreover, the effect of LLE treatments on mycelia growth, spore germination, morphology alteration, and membrane lipid peroxidation of *P. digitatum* was explored to provide a mechanistic overview of antifungal activity.

## Materials and Methods

### Chemicals and Preparation of Loquat Leaf Extract

Ethanol, n-butanol, glucose, and agar powder were procured from Sinopharm Chemical Reagent Co., Ltd. (Shanghai, China). Air-dried loquat leaves (purchased from Zhangshu medicinal materials market and authenticated as leaves of *Eriobotrya japonica* Lindl) were finely pulverized and extracted with 50% ethanol (100 g of dry powder per 1 L) at 60°C for 1 h. The extracts were then filtered and concentrated under reduced pressure using a rotary evaporator (Rotavapor R-3, Buchi, Flawil, Switzerland) at 45°C. After that, the extracts were suspended in water and sequentially extracted with ethyl acetate and n-butanol. The n-butanol extract was filtrated, combined, and evaporated to obtain dried LLE. The dried LLE was stored in a cryovial at −20°C until further analysis.

### Fungal Preparation

The phytopathogenic fungi *P. digitatum* (CGMCC 3.15410) and *P. italicum* (CGMCC 3.4040) were bought to the laboratory from the center of China General Microbiological Culture Collection (Beijing, China). *G. citri-auranti* was provided by the Department of Plant Protection in Jiangxi Agricultural University (Nanchang, China) and identified by Prof. Junxi Jiang. *D. citri* was isolated from decayed citrus fruits with a representative disease symptom of *Phomopsis* stem-end rot and identified by DNA sequencing (Qingke Biotech, Changsha, China). Each fungus was cultured on a potato dextrose agar (PDA) medium (leaching solution of potato 200 g, glucose 20 g, agar powder 20 g, and deionized water, 1 L) at 25 ± 1°C for a reactivation period of 7 days. The spore suspension of *P. digitatum* was prepared based on the protocol of previous studies (1, 14).

### Antifungal Activity of LLE Against *P. digitatum*

#### Evaluation of MIC and MFC

Both the minimal inhibitory concentration (MIC) and the minimal fungicidal concentration (MFC) of loquat leaf extract (LLE) against *P. digitatum* were evaluated *in vitro* using the method of Chen et al. (15). The dried LLE was dissolved with double-distilled water (ddH_2_O) and mixed with a PDA medium; then, the final concentrations of LLE ranged from 0 to 1.25 mg/ml per disc. Using a punch to transfer the mycelial plugs at the central of the PDA dish, After the cultivation of 25°C for 2 and 6 days to evaluate its antifungal activity. The values of MIC and MFC were defined as the lowest concentration of LLE that inhibited *P. digitatum* growth after incubation for 2 and 6 days, respectively.

#### Mycelium Growth

The antifungal activity of loquat leaf extract (LLE) against *P. digitatum* was determined using the protocol of Chen et al. (14) and defined as percent growth inhibition. Briefly, the discs (5 mm) of *P. digitatum* were placed in the center of Petri dishes containing 20 ml PDA with different LLE concentrations of 0 (set as the control), 0.156, 0.313, 0.625, 1.25, and 2.5 mg/ml. After incubation at 25°C for 6 days, the colony diameters (mm) of the control and LLE treated fungal growth were examined using a vernier caliper. The data were duplicated three times and expressed as the means ± standard deviation (SD).

#### Spore Germination

The spore germination assay determined the antifungal ability of LLE against *P. digitatum* following the method of Tao et al. (16). Briefly, the LLE was dissolved in potato dextrose broth (PDB) to acquire five different concentrations of 0 (set as the control), 0.156, 0.313, 0.625, and 1.25 mg/ml. Subsequently, 5 μl of *P. digitatum* spore suspension (1 × 10^6^ CFU/ml) was added to different LLE-treated slides. After 13 h of incubation at 25°C, nearly 100 spores per replicate were observed using an optical microscope, and the inhibitory germination rate of *P. digitatum* spore was estimated using the following formula:

$$\begin{array}{l} \text{IGR}=\frac{\text{G}{\text{S}}_{\text{C}}-\text{G}{\text{S}}_{\text{LLE}}}{\text{G}{\text{S}}_{\text{C}}}\times 100 \end{array}$$

where IGR is inhibitory germination rate, GS_C_ is the mean amount of germinated spore in the control slide, and GS_LLE_ is the mean amount of germinated spore in the LLE-treated slide. The experiments were conducted twice with three replicates for each treatment.

### Scanning Electron Microscopy Observations

The effect of LLE on microscopic morphological alterations in *P. digitatum* spore was observed by SEM (15). *P. digitatum* spores, which included the control and LLE-treated samples, were washed with a PBS buffer three times and subsequently fixed with 2.5% (v/v) glutaraldehyde at 4°C. After fixation for 48 h, the samples were rinsed three times in distilled water for 20 min and then dehydrated with sequential graded cold ethanol (30, 50, 70, and 90%) for 20 min, and finally with absolute ethanol for 45 min. The specimens were then sputter-coated with gold and observed with a scanning electron microscope (FEI Quanta 250 FEG, Hillsboro, OR, United States).

### Assay of LLE on Cell Membrane Permeability of *P. digitatum*

The effects of LLE on the cell membrane permeability of *P. digitatum* were investigated by assaying extracellular conductivity, cell lysis rate, and leakages of protein and nucleic acids. Briefly, 200 μl of a *P. digitatum* suspension was evenly added in 100 ml of PDB and incubated in a shaker for 2 days. About 2 g of fresh mycelium was re-suspended in 50 ml PDB having different LLE concentrations of 0 (set as the control), MIC (0.625 mg/ml), and MFC (1.25 mg/ml). The samples were taken at various time intervals (0, 30, 60, 90, 120, and 240 min). Extracellular conductivity was detected as reported by Tao et al. (16) with a conductivity meter (model ST3100C, Ohaus Co., Parsipanny, NJ, United States). Cell lysis rate was evaluated according to the method of Chen et al. (1). Leakages of protein and nucleic acid from *P. digitatum* hyphae were determined based on the method described by Huang et al. (17) to measure the optical density (OD) of the supernatant at 260 and 280 nm. The experiments were conducted twice with three replicates for each treatment.

### Measurement of Loss in Intracellular Constituents

The loss in intracellular constituents into the supernatant was measured according to the method described previously (18), with minor modifications. Soluble protein, reducing sugar, total lipid, and ergosterol contents in both the supernatants and the hyphae of *P. digitatum* were measured using bovine serum albumin standards, glucose, and cholesterol, respectively. The experiments were conducted twice with three replicates for each treatment.

### Propidium Iodide Fluorescence Staining and Membrane Lipid Peroxidation

The effect of LLE on the plasma membrane integrity of *P. digitatum* was determined following the method described by Xin et al. (19). Briefly, *P. digitatum* hyphae were treated with LLE at 0 and 1.25 mg/ml (MFC) for 120 min. Then, the control and LLE-treated mycelia were dyed with propidium iodide (PI) at 25°C in the dark. After staining for 30 min, the control and LLE-treated mycelia were washed three times with PBS and observed with a Ni-U fluorescence microscope (Nikon Corporation, Tokyo, Japan).

The lipid peroxidation of *P. digitatum* cell was quantitatively determined in terms of malondialdehyde (MDA) content using the thiobarbituric acid method described by Pasquariello et al. (20), and MDA content was calculated using the equation below:

$$\begin{array}{l} \text{lipid peroxidation }=\;[\text{6}.45\;\times \;(\text{OD}532\;-\text{ OD}600)\;-\;0.559\;\\ \;\times \text{ OD}450]\times \text{ Vt}/\text{FW} \end{array}$$

where Vt is the total volume of the extract (ml), and FW is the frozen weight of *P. digitatum* (g).

The experiments were conducted twice with three replicates for each treatment.

### Assays of LLE on the Activities of β-1,3-Glucanase and Alkaline Phosphatase

The 2-day-old mycelia from 100 ml PDB were collected and resuspended in 50 ml PDB with LLE at various concentrations (0.625, and 1.25 mg/ml). β-1,3-glucanase (β-Glu) activity, after exposure to LLE for 0, 30, 60, 90, 120, and 240 min, was measured according to the previously developed method (1). According to the instructions of the manufacturer, the extracellular AKP activity of *P. digitatum* hyphae after exposure to LLE treatments was determined using a commercial AKP kit (Jiancheng Bioengineering Research Institute Co., Nanjing, China) (21), and enzyme activities were expressed as U/mg prot. The experiments were conducted twice with three replicates for each treatment.

#### Assays of LLE on ATP, ADP, and AMP Content

The hyphae of *P. digitatum* were incubated according to the method for both β-Glu and AKP activity assays mentioned above. ATP, ADP, and AMP contents were determined following the method of Zheng et al. (22), and expressed as mg·kg^−1^ on a dry weight basis. The value of energy charge (EC) was calculated using the formula below:

$$\begin{array}{l} \text{EC}=\left(\left[\text{ATP}\right]+1/2\;\times \;\left[\text{ADP}\right]\right)/\left(\left[\text{ATP}\right]+\left[\text{ADP}\right]+\;\left[\text{AMP}\right]\right) \end{array}$$

The experiments were conducted twice with three replicates for each treatment.

### Statistical Analysis

Comparisons of data from different groups were analyzed by one-way analysis of variance (ANOVA) at 5% significance level with the SPSS 22.0 software (SPSS Inc. Chicago, IL, United States). The levels of significance were assessed as significant (*p* < 0.05) and highly significant (*p* < 0.01) using Prime and Excel 2010 software.

## Results

### MIC and MFC

The treatments of LLE completely inhibited the mycelial growth of *P. digitatum, P. italicum*, and *D. citri* at the concentration of 0.625 mg/ml when incubated for 2 days. LLE (1.25 mg/ml) completely inhibited mycelial growth on the sixth day (Table 1). The results revealed that the MIC and MFC values of LLE against *P. digitatum, P. italicum*, and *D. citri* were 0.625 and 1.25 mg/ml, respectively. Moreover, LLE at the concentration of 0.625 and 2.5 mg/ml completely inhibited *G. citri-auranti* growth on the second and sixth days of incubation, respectively (Table 1). Hence, the MIC and MFC values of LLE against *G. citri-auranti* were 0.625 and 2.5 mg/ml, respectively.

**Table 1:** Minimum inhibitory concentration (MIC) and minimum fungicidal concentration (MFC) of loquat leaf extract (LLE) against *P. digitatum, P. italicum, D. citri*, and *G. citri-auranti*.

**Concentration (mg/ml)**	***P. digitatum***	***P. italicum***	***D. citri***	***G. citri-auranti***
MIC	0.625	0.625	0.625	0.625
MFC	1.25	1.25	1.25	2.50

### Effects of LLE on Mycelial Growth and Spore Germination of *P. digitatum*

**Figure 1:**
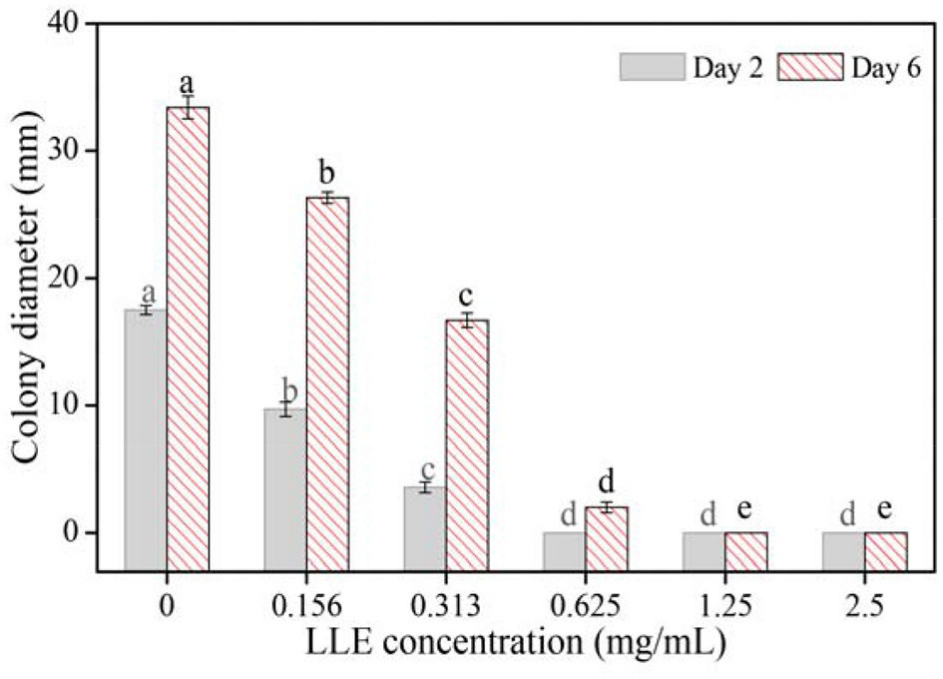
Mycelial growth of *P. digitatum* on potato dextrose agar (PDA) exposure to loquat leaf extract (LLE) after 2 and 6 days. Different letters above the columns of the same day represent significant differences (*p* < 0.05) in colony diameter among the LLE concentrations.

Different LLE concentrations showed potent inhibitory effects on the mycelial growth of *P. digitatum* on the PDA culture, compared with the control group (Figure 1). After 2 days, the diameter of the colony decreased significantly (*p* < 0.05) with increased LEE concentration, and the extension of the *P. digitatum* colony disappeared completely at LLE concentrations ≥0.625 mg/ml (Figure 1). Besides, the mycelial growth rate, after 48 h of culture with LLE concentrations of 0.625 and 1.25 mg/ml, decreased to 57.5 and 20.3%, respectively, relative to the mycelial growth in the control group (Figure 1). Furthermore, the mycelial growth of *P. digitatum* was negligible over the incubation period when treated with LEE concentrations of ≥1.25 mg/ml.

As shown in Table 2, a significant inhibitory effect of LLE on the spore germination of *P. digitatum* in the PDB medium was seen in a dose-dependent manner, where higher LLE concentration resulted in a lower spore germination rate. LLE at a concentration of 0.625 mg/ml could significantly (*p* < 0.05) suppress the spore germination of *P. digitatum* by 78.95 ± 0.41% compared with the control group (Table 2); whereas at an LLE concentration of 0.156 mg/ml, only 17.62 ± 0.45% of inhibitory percentage was recorded. As the LLE concentration increased up to 1.25 mg/ml, *P. digitatum* spore germinated rate was <5%. The linear regression of the inhibitory percentage of *P. digitatum* (Y) on the log-transformed LLE-treated concentrations (X) was determined as Y = 1.75X−1.491, R^2^ = 0.987, with the half-maximal effective concentration (EC_50_ refers to the LLE dose causing 50% inhibition of the spore germination) of LLE against *P. digitatum* being 0.317 mg/ml.

**Table 2:** Effect of LLE on spore germination of *P. digitatum*.

**Concentration (mg/ml)**	**Spore germination rate (%)**	**Inhibitory percentage (%)**
1.25	2.13 ± 0.81	97.79 ± 0.84a
0.625	20.27 ± 0.35	78.95 ± 0.41b
0.313	54.50 ± 0.46	43.40 ± 0.62c
0.156	79.33 ± 0.31	17.62 ± 0.45d
0 (control)	96.3 ± 0.26	0.00 ± 0.00e

*Different letters (a–e) represent significant differences (p < 0.05)*.

### Effect of LLE on Microscopical Morphology of *P. digitatum* by SEM

**Figure 2:**
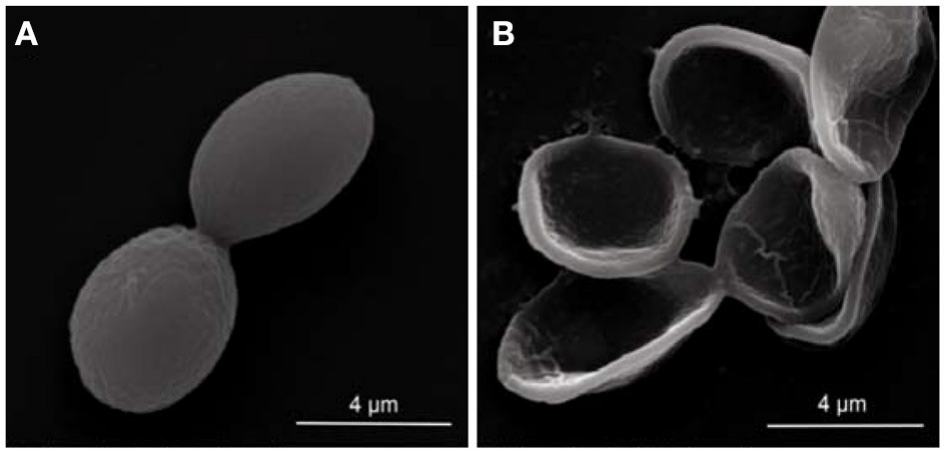
Scanning electron microscopy (SEM) observation (× 25,000 magnification) of the morphology alteration of *P. digitatum* spores exposed to 1.25 mg/ml (minimum fungicidal concentration, MFC) loquat leaf extract (LLE): **(A)** untreated spores and **(B)** LLE-treated spores.

The scanning electron microscopy images showed that the microscopical morphology of the *P. digitatum* spores was severely affected by LLE treatment. The untreated spores had an engorged globular shape, with a smooth surface (Figure 2A). However, LLE treatment at the MFC (1.25 mg/ml) altered the microscopical morphology of the *P. digitatum* spores, including sunken surface, loss of linearity, and malformation (Figure 2B).

### Effect of LLE on Cell Membrane Permeability of *P. digitatum*

**Figure 3:**
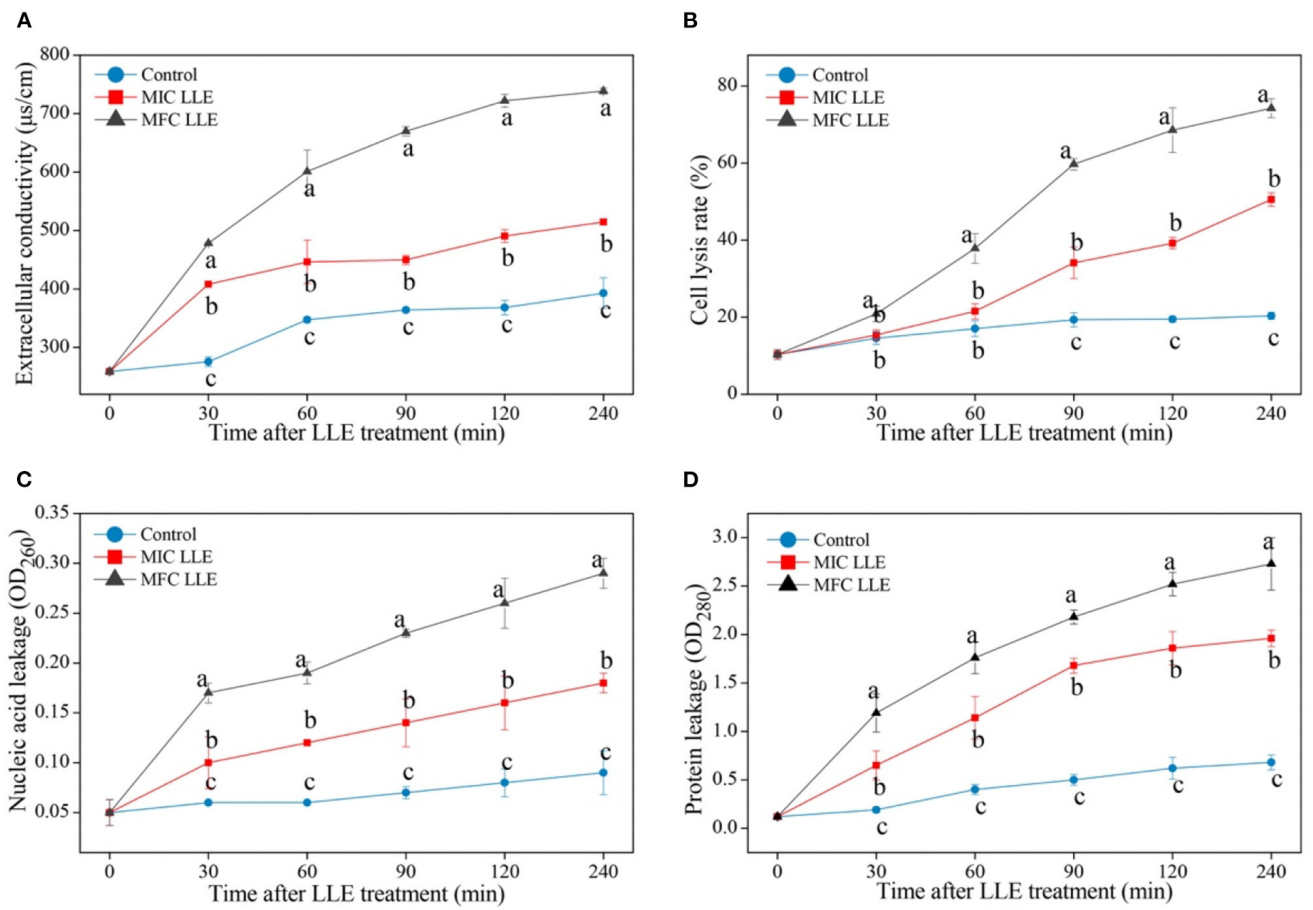
Effect of loquat leaf extract (LLE) on cell membrane permeability of *P. digitatum*. **(A)** Change in extracellular conductivity of *P. digitatum* exposed to LLE treatment; **(B)** change in cell lysis rate of *P. digitatum* exposed to LLE treatment; **(C)** change in nucleic acid leakage of *P. digitatum* exposed to LLE treatment; and **(D)** change in protein leakage of *P. digitatum* exposed to LLE treatment. Different letters above the lines represent significant differences (*p* < 0.05) among treatments simultaneously.

The effect of LLE treatment on the cell membrane permeability of *P. digitatum* is presented in Figure 3. The extracellular conductivity in the 1.25 mg/ml LLE-treated *P. digitatum* cell suspension increased from 259.1 ± 1.03 μs/cm to 722.2 ± 11 μs/cm after 120 min of incubation (Figure 3A, *p* < 0.05). The cell lysis rate in LLE-treated *P. digitatum* suspensions significantly increased (*p* < 0.05), whereas the control group remained stable. After 240 min of incubation, the cell lysis rate in the *P. digitatum* suspension treated with 0.625 and 1.25 mg/ml LLE was 50.54 ± 1.71 and 74.23 ± 2.54%, respectively, compared with 20.35 ± 0.92% in the control group (Figure 3B). As illustrated in Figures 3C,D, LLE treatment significantly induced the leakages of nucleic acid and protein in *P. digitatum* hypha (*p* < 0.05). After 240 min of incubation, both nucleic acid leakage and protein leakage in *P. digitatum* suspensions treated with 1.25 mg/ml LLE were 3.22 times and 4.01 times higher than that of the control. These results might indicate that cell membrane permeability was likely to be one of the vital antifungal mechanisms for LLE in *P. digitatum* growth.

**Figure 4:**
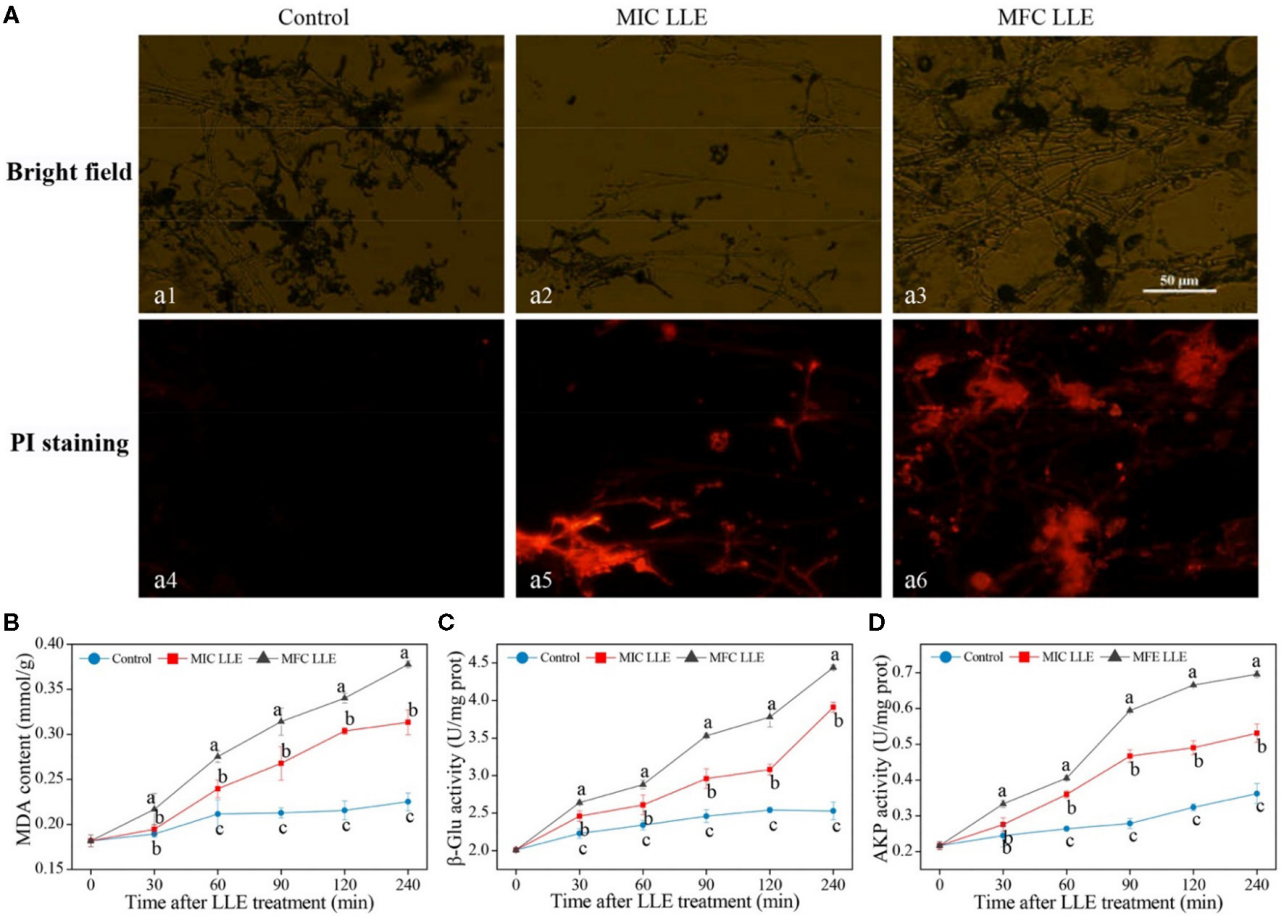
Effect of loquat leaf extract (LLE) on cell membrane permeability and lipid peroxidation of *P. digitatum*. **(A)** Propidium iodide (PI) staining, a_1_, a_4_: control treatment (0 mg/ml LLE.); a_2_, a_5_: minimum inhibitory concentration (MIC treatment) (0.625 mg/ml LLE); a_3_, a_6_: minimum fungicidal concentration (MFC) treatment (1.25 mg/ml LLE); **(B)** change in malondialdehyde (MDA) content of *P. digitatum* exposed to LLE treatment; **(C)** change in the β-1,3-glucanase (β-Glu) activity of *P. digitatum* exposed to LLE treatment; **(D)** change in the alkaline phosphatase (AKP) activity of *P. digitatum* exposed to LLE treatment. Different letters above the lines represent significant differences (*p* < 0.05) among treatments at the same time after treatment.

The fluorescence microscopy images of *P. digitatum* hypha treated with MIC and MFC LLE are shown in Figure 4A, and are consistent with the cell membrane permeability results described above. No visible red fluorescence was observed in the control *P. digitatum* hypha after 30 min of incubation. In contrast, robust red fluorescence was observed in the MIC and MFC LLE-treated samples, respectively.

### Effect of LLE on Intracellular Constituents of *P. digitatum*

**Figure 5:**
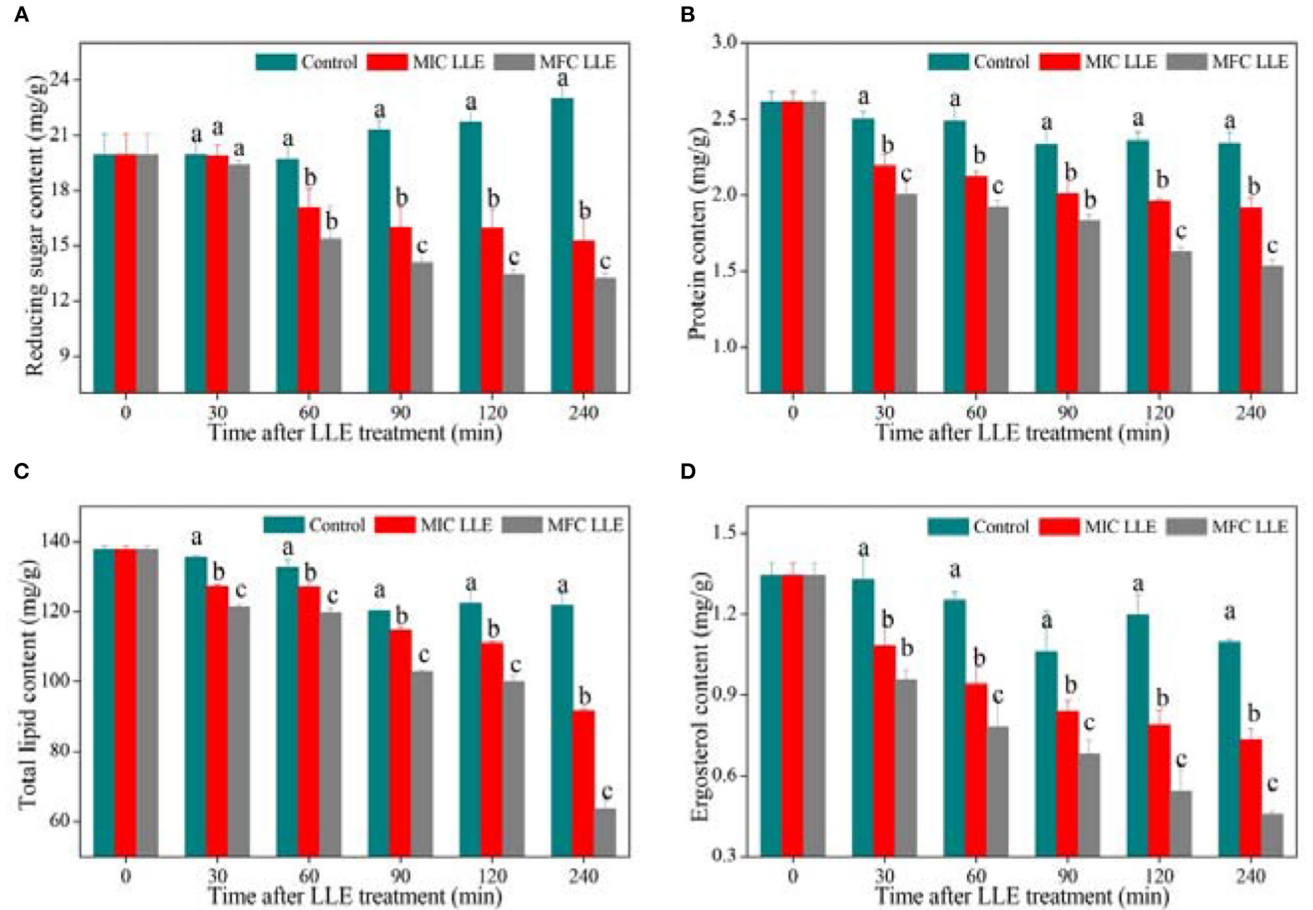
Effect of loquat leaf extract (LLE) on intracellular constituents of *P. digitatum*. **(A)** Change in reducing sugar content of *P. digitatum* exposed to LLE treatment; **(B)** change in protein content of *P. digitatum* exposed to LLE treatment; **(C)** change in total lipid content of *P. digitatum* exposed to LLE treatment; and **(D)** change in ergosterol content of *P. digitatum* exposed to LLE treatment. Different letters above the columns represent significant differences (*p* < 0.05) among treatments simultaneously after treatment.

Changes in intracellular reducing sugar, protein, total lipid, and ergosterol contents of *P. digitatum* exposed to LLE treatment at 0 mg/ml, MIC, and MFC are presented in Figure 5. A significant difference in intracellular reducing sugar content was observed after 60 min of exposure and gave a declining trend with increasing LLE concentrations (Figure 5A). After 120 min of incubation, the reducing sugar content in *P. digitatum* hypha was 15.95 ± 1.05 and 13.45 ± 0.27 mg/g at LLE concentrations 0.625 and 1.25 mg/ml, respectively, which was significantly lower than that in the control samples (21.7 ± 0.72 mg/g, *p* < 0.05). As demonstrated in Figure 5B, the protein content of control *P. digitatum* hypha was found to maintain a stable level during an incubation period of 0–240 min. In contrast, in the LLE-treated groups, the protein content significantly decreased. For instance, at 240 min after the treatment, the intracellular protein content in *P. digitatum* hypha treated with the MIC and MFC LLE was 17.95 and 34.62% lower than that of the control, respectively. In addition, the total lipid content in LLE-treated *P. digitatum* hypha decreased with increasing exposure time. In contrast, the total lipid content in the MIC and MFC LLE-treated samples after 240 min of incubation was 91.65 ± 0.62 and 63.69 ± 2.27 mg/g, respectively, which was significantly lower than that in the control group (121.78 ± 3.64 mg/g, *p* < 0.05, Figure 5C). Similarly, a continuous decrease in the ergosterol content of *P. digitatum* hypha after LLE treatment was observed throughout the whole incubation period, whereas the ergosterol content in the control group remained stable (Figure 5D). After 120 min of incubation, the ergosterol content in *P. digitatum* hypha treated with the MIC and MFC LLEs were 0.79 ± 0.06 and 0.54 ± 0.08 mg/g, respectively, which was significantly lower (*p* < 0.05) than that observed in the control group (1.2 ± 0.08 mg/g).

### Effect of LLE on Lipid Peroxidation of *P. digitatum*

The effect of loquat leaf extract LLE treatment on the malondialdehyde (MDA) content of *P. digitatum* hypha is shown in Figure 4B. After 60 min of LLE treatment, a striking difference between the control and LLE-treated samples was observed, with the intracellular MDA content in the MIC and MFC LLE-treated groups being 2.61 ± 0.13 and 2.88 mmol ± 0.06 mmol/g, respectively, which was significantly higher than that in the control group (2.34 ± 0.06 mmol/g, *p* < 0.05). As shown in Figure 4B, the β-Glu activity in the control *P. digitatum* hypha was found to maintain a stable level during an incubation period of 0–240 min. In contrast, it rapidly increased in the LLE-treated groups. For instance, at 120 min of incubation, the β-Glu activity in *P. digitatum* hypha treated with the MIC and MFC LLEs were 1.47 times and 1.92 times higher than that of the control, respectively (Figure 4C, *p* < 0.05). The AKP activity of the MIC and MFC LLE-treated *P. digitatum* hypha was.27 ± 0.019 and 0.31 ± 0.015 U/mg, respectively, at 90 min after treatment, which was higher (*p* < 0.05) than that in the control samples. As the treatment time increased to 240 min, the AKP activity of *P. digitatum* hypha treated with LLE at MIC and MFC were 1.39 times and 1.68 times, respectively, higher than that of the control group (Figure 4D, *p* < 0.05).

### Effect of LLE on ATP, ADP, and AMP Contents of *P. digitatum*

**Figure 6:**
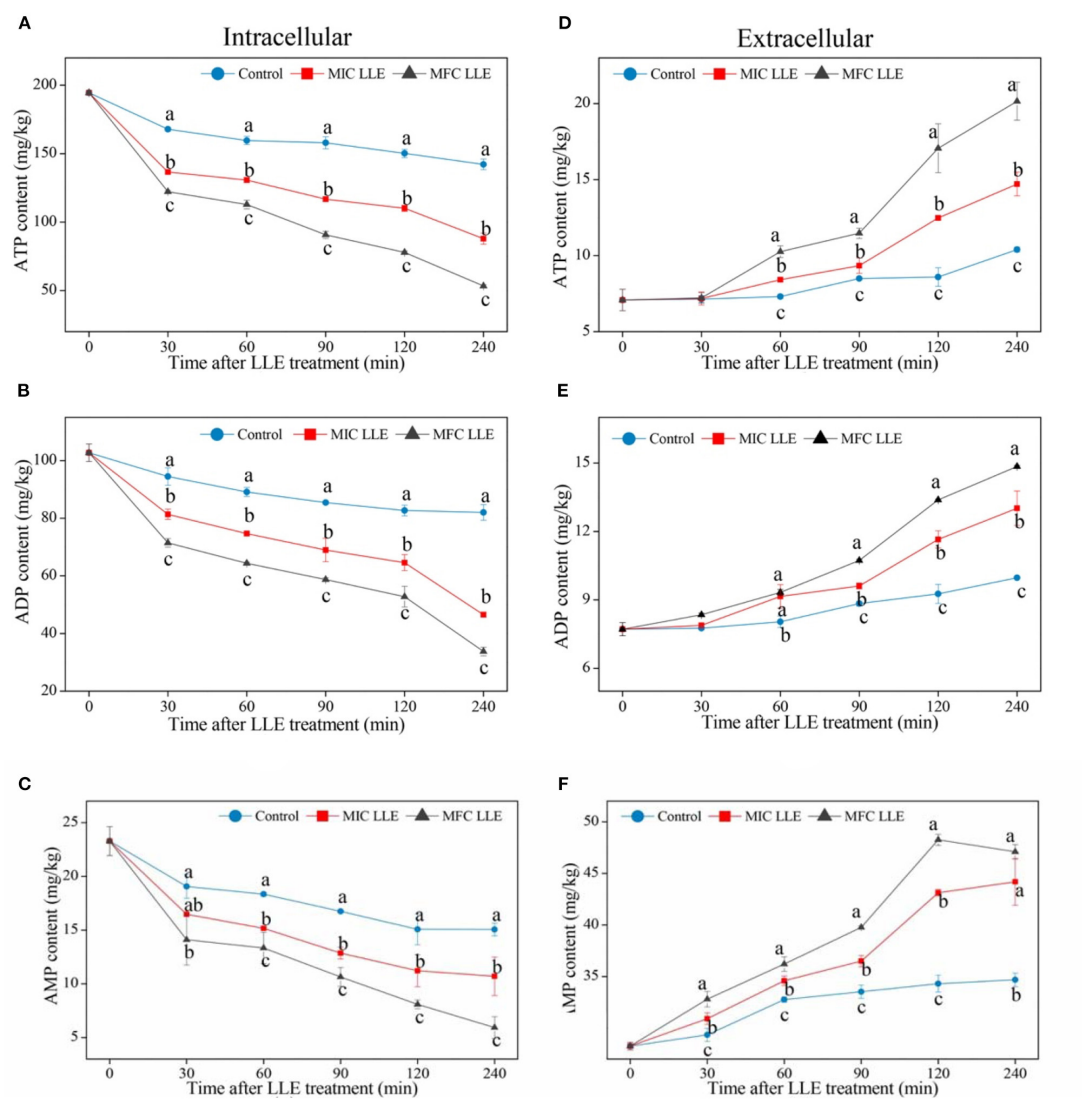
Effect of loquat leaf extract (LLE) on adenosine triphosphate (ATP) **(A,D)**, adenosine diphosphate (ADP) **(B,E)**, and adenosine monophosphate (AMP) **(C,F)** contents in intracellular (left) and extracellular (right) *P. digitatum*. Different letters above the lines represent significant differences (*p* < 0.05) among treatments at the same time after treatment.

The effects of LLE treatment on the ATP, ADP, and AMP contents in *P. digitatum* hypha are presented in Figure 6. As shown in Figure 6A, the ATP content of *P. digitatum* hypha treated with LLE at MIC and MFC is 110.1 ± 2.25 and 77.9 ± 1.05 mg/kg, respectively, which is much lower than that in the control samples after 120 min of incubation (150.2 ± 3.03 mg/kg), indicating that the ATP supply of *P. digitatum* hypha is hindered by LLE treatment. Figure 6D shows the effect of ATP content release when the *P. digitatum* cell suspension is treated with LLE at MIC and MFC. The ATP content of the *P. digitatum* cell suspension treated with MFC LLE was dramatically increased after 120 min of exposure, which was significantly higher (*p* < 0.05) than that in MIC LLE (36.7%) or the control (98.6%). Similarly, both the ADP and AMP contents of *P. digitatum* hypha significantly decreased with the MIC and MFC LLE treatments (Figures 6B,C, *p* < 0.05). In comparison, the release of ADP and AMP of the *P. digitatum* cell suspension significantly increased with the MIC and MFC LLE treatment (Figures 6E,F, *p* < 0.05). Those results suggested that the intracellular energy source of *P. digitatum* was infiltrated into the cell suspension after LLE treatment. Thus, the extracellular ATP, ADP, and AMP levels in the *P. digitatum* cell suspension exposed to LLE treatment was much higher than those of the control.

## Discussion

As we all know, loquat leaves have been proven to have good anti-inflammatory effects (23). Similarly, Cynanchum atratum (24), Mentha (25), and Ficus hirta Vahl (26, 27) showed anti inflammatory effects, along with strong antifungal effects [Cynanchum atratum (28), Ficus hirta Vahl (1, 29, 30), Mentha essential oil (31, 32)]. Therefore, the antifungal effect of loquat leaves needs to be verified, and then, its natural antifungal substances should be studied to provide the basis for developing new pesticides-botanical fungicides. In this study, the antifungal effects of LLE against *P. digitatum, P. italicum, D. citri*, and *G. citri-auranti* were evaluated *via* the agar dilution culture method (33). The results showed that the MIC value of LLE against these four fungi was 0.625 and 1.25 mg/ml of MFC against *P. digitatum, P. italicum*, and *D. citri* (Table 1). Furthermore, *P. digitatum* mycelial growth and spore germination inhibition were LLE dose-dependent (Figure 1; Table 2). These results showed that LLE has a strong inhibitory effect on *P. digitatum* and has a potential application prospect.

The antifungal mechanism of loquat leaf extract (LLE) was directly reflected by the morphological changes in *P. digitatum* spores, causing surface shrinkage, empty spores, and overflow of cell contents, which damaged the cell wall and cell membrane structure. The cell membrane lysis of *P. digitatum* was seen by changes in electrical conductivity (Figure 3A), nucleic acid leakage (Figure 3C), and protein leakage (Figure 3D). Thus, LLE caused disorder in the transmembrane electromotive force of the cell membrane, thereby damaging the function of the cell membrane, and the cell wall of *P. digitatum*.

The fungal cell wall is composed of polysaccharide macromolecules, such as dextran and chitin. The cell wall plays a vital role in maintaining normal cell morphology, controlling the transport of vital substances, and providing a defensive mechanism. β-1, 3-glucanase hydrolyzes β-1,3-glucans, which are the primary polymers within the fungal cell wall. AKP is also a hydrolytic enzyme that causes dephosphorylation from nucleotides, proteins, alkaloids, and other molecules. In this study, the β-1, 3-glucanase activity (Figure 4C) of the mycelium and the AKP activity (Figure 4D) of the extracellular after LLE treatment was increased. The LLE was supposed to exert its antifungal activity by causing physical damage to the cell wall of *P. digitatum* and also by increasing the cell wall degradation enzymatic activity, thereby destroying cell wall biosynthesis and integrity, leading to cytoplasmic collapse and eventually causing cell death.

The cell membrane is a selective semi-permeable membrane whose integrity, fluidity, and selective permeability can control the movement of various substances, which are vital for microbial growth and pathogenicity. Moreover, the PI fluorescence staining results (Figure 4A) showed that the PI entered the cell and intercalated into the DNA, and emitted a large amount of red fluorescence, reflecting loss of cell wall integrity. These results were also shown in other studies (34). Polyphenols and triterpenes are abundant in the loquat plant (35), which may be involved in the impairment of the cell membrane (36, 37), thereby exerting antifungal activity. Besides, in physiological metabolism, it has been reported that lipid peroxidation is also part of the antifungal mechanism (38). MDA is the main product of lipid peroxidation, which can be used as an index to measure the damage of membrane lipid peroxidation. In this study, the mycelium MDA content of *P. digitatum* treated with loquat leaves was remarkably higher than that of the untreated group (Figure 4B). This illustrated that the extract could accelerate the membrane lipid peroxidation of *P. digitatum*, cause fluidity of the cell membrane, and increase cell wall permeability.

Lipid and ergosterol are essential components of the cell membrane (39). Ergosterol plays a magnificent role in maintaining cell viability, membrane integrity, and fluidity (40). Xin et al. (19) showed that the antifungal mechanism of Baiwei extract was due to its capability to destroy the integrity of the cell membrane, which is mostly influenced by the content of total lipid and ergosterol in the cell membrane. The effect of extract treatment on cell membrane composition was studied by measuring total lipid and ergosterol content in the cell membrane. As shown in Figure 5C, the LLE extract can reduce total lipid content in the membrane in a dose-dependent manner, affecting the stability of the cell membrane and increasing its fluidity. Helal et al. (40) proposed that a decrease in lipids will hinder the transport of fat-soluble substances and destroy cell-selective permeability. Figure 5D shows that LLE can significantly reduce ergosterol content, indicating that LLE could act on ergosterol and inhibit its synthesis. The decrease in lipid and ergosterol content reflected the irreversible damage of the cell membrane (40), thus we strongly postulated that the cell membrane might be the site of the LLE target.

Cells need nutrients to produce the energy for their growth and activities; hence nutrients are essential to cells. However, after the destruction of the cell membrane, pathways for the synthesis and transport of substances in cells may also be affected, thus preventing normal physiological metabolism of cells (41). The results of this study showed that after treatment with LLE, the contents of reducing sugar and soluble protein of *P. digitatum* cell suspensions were markedly higher than those of the untreated group (Figure 5). This may be due to increased membrane permeability with LLE, which causes an increase in the outflow of reducing sugar and soluble protein.

In the process of physiological metabolism, energy metabolism is also one of the essential metabolic pathways. ATP is the center of energy storage and utilization, and biochemical reactions must ensure various activities. Therefore, the change in its content in cells can directly affect the physiological activities of cells (42). Moreover, the ratio of various adenosine phosphates (ATP, ADP, and AMP) reflects cell energy charge (EC). Many metabolic activities in cells depend on energy charge changes, such as glycolysis, tricarboxylic acid cycle, electron transport system, and oxidative phosphorylation (43). Therefore, the extract of loquat leaves can significantly affect the changes in intracellular energy substances in *P. digitatum*, resulting in decreased intracellular ATP, ADP, and AMP content (Figures 6A–C). Moreover, the extract of loquat leaves leads to increased extracellular energy substances (Figures 6D–F). These phenomena may hinder the ATP synthesis pathway; hence the intracellular ATP has a significant downward trend, followed by ADP and AMP. The decrease in the intracellular synthesis of storage molecules by LLE leads to cell apoptosis and thus exerted its antifungal activity.

## Conclusions

The current study revealed a strong antifungal activity of LLE against the citrus postharvest pathogen *P. digitatum*. The LLE exhibited strong antifungal activity against *P. digitatum*, with a minimum inhibitory concentration of 0.625 mg/ml and a minimum fungicidal concentration of 1.25 mg/ml, respectively. Sunken surface and malformation occurred at the LLE-treated *P. digitatum* spores. Besides, a higher increase of cell death was observed in propidium iodide (PI) fluorescent staining in the presence of LLE. Furthermore, LLE treatment induced a significant decline of the intracellular energy substances (ATP, ADP, and AMP) content during the entire treatment period. Those results manifest that the antifungal activity of LLE against *P. digitatum* can be attributed to the derangement of cell membrane permeability and the disordered energy metabolism. The present study is proving a new mechanism of LLE extract against *P. digitatum*, which could be further tested at molecular level along with field trial. The study results could be helpful to use LLE extract as a natural antifungal product for preventing the growth and activity of *P. digitatum* and thus prevented the postharvest citrus fruit losses in a more sustainable manner.

## Data Availability Statement

The raw data supporting the conclusions of this article will be made available by the authors, without undue reservation.

## Author Contributions

CW and JC: conceptualization, resources, supervision, and project administration. YS, CC, NC, and RY: methodology and investigation. YS and CC: software, validation, formal analysis, data curation, and writing—original draft preparation. CW, İK, VO, KR, and JC: writing—review and editing. İK, YS, and CC: visualization. CW: funding acquisition. All authors have read and agreed to the published version of the manuscript.

## Conflict of Interest

The authors declare that the research was conducted in the absence of any commercial or financial relationships that could be construed as a potential conflict of interest.

## Publisher's Note

All claims expressed in this article are solely those of the authors and do not necessarily represent those of their affiliated organizations, or those of the publisher, the editors and the reviewers. Any product that may be evaluated in this article, or claim that may be made by its manufacturer, is not guaranteed or endorsed by the publisher.
